# Lactation Curve Pattern and Prediction of Milk Production Performance in Crossbred Cows

**DOI:** 10.1155/2014/814768

**Published:** 2014-07-13

**Authors:** Suresh Jingar, R. K. Mehla, Mahendra Singh, A. K. Roy

**Affiliations:** ^1^National Dairy Research Institute, Karnal 132 001, India; ^2^KVK, Chittorgarh, Rajasthan 312001, India

## Abstract

Data pertaining to 11728 test-day daily milk yields of normal and mastitis Karan Fries cows were collected from the institute herd and divided as mastitis and nonmastitis and parity-wise. The data of lactation curves of the normal and mastitis crossbred cows was analyzed using gamma type function. FTDMY in normal and mastitis cows showed an increasing trend from TD-1 to TD-4 and a gradual decrease (*P* < 0.01) thereafter until the end of lactation (TD-21) in different parities. The FTDMY was maximum (peak yield) in the fourth parity. Parity-wise lactation curve revealed a decrease in persistency, steeper decline in descending slope (*c*), and steeper increase in ascending slope (*b*) from 1st to 5th and above parity. The higher coefficient of determination (*R* ^2^) and lower root mean square error (RMSE) indicated goodness and accuracy of the model for the prediction of milk prediction performance under field conditions. Clinical mastitis resulted in a significantly higher loss of milk yield (*P* < 0.05). The FTDMY was maximum (*P* < 0.05) in the fourth parity in comparison to the rest of parity. It is demonstrated that gamma type function can give the best fit lactation curve in normal and mastitis infected crossbred cows.

## 1. Introduction

Lactation curve provides valuable information about the pattern of milk production during lactation. It also depicts summary of the pattern of milk yield determined by the biological efficiency of the cow [1]. The cost of milk production depends to a large extent on the persistency of lactation, that is, the rate of decline in production after peak milk yield. High persistency is associated with a slow rate of decline in milk production, whereas low persistency is associated with a rapid rate of decline in milk yield. In general declining rate of milk production is about 7% per month after the peak yield [2]. Estimates of heritabilities for milk yield and persistency traits in HF cows have been reported [3, 4]. The lactation curve models have been used to predict the milk yield at any point of the lactation [5, 6]. This property of the model can prove beneficial in case of incomplete lactation records. Various models have been tried by different researchers to fit the lactation curve in indigenous as well as exotic cattle [7–10]. Further getting the test-day milk yield information from the field conditions is not easy and there is every chance of missing the data due to certain inevitable circumstances. In such case mathematical models may prove beneficial for prediction of milk production performance using. In view of this the lactation curve was composed for the normal vis-à-vis mastitis cows using gamma type function during different parity to find out the accuracy of model in predicting the milk yield with minimum error in normal and mastitis crossbred cows.

## 2. Materials and Methods

The data on 11728 fortnightly test-day milk yields (FTDMYs) of 733 Karan Fries cows for a period of 12 years (2000–2011) was collected from livestock farm of National Dairy Research Institute, Karnal, India. The climate of the farm is subtropical in nature with the lowest temperature reaching 2°C during winter and the highest temperature up to 45°C in the summer months. The annual rainfall is about 760 to 960 mm, out of which most of the rainfall is received during the months of July and August. The relative humidity ranges from 41% to 85%. The amount of milk recorded in 24-hour duration on any particular day is called the test-day (TD) milk yield and is expressed in kilograms. The fortnightly test-day intervals of 15 days were considered starting from days 5, 20, 35, 50, 65, 80, 95, 110, 125, 140, 155, 170, 185, 200, 215, 230, 245, 260, 275, 290, and 305 of lactation. Twenty-one (21) fortnightly test-day milk yield (FTDMY) records were considered per lactation (from the 6th to the 305th day of lactation). The data was grouped as mastitis (cows suffering from clinical mastitis) and nonmastitis (normal cows). Statistical analysis of data was carried out using gamma type function [11] with the following equation:

(1) $$\begin{array}{c} Y_{t}=at^{b}e^{-ct}, \end{array}$$

where *Y*_*t*_ is the average daily yield in the *t*th fortnight, *a* is the initial milk yield after calving, *b* is the ascending slope parameter up to the peak yield, and *c* is the descending slope parameter.

The constants were derived by solving the above equation after transformation on the log scale:

(2) $$\begin{array}{c} \ln\left(Y_{t}\right)=\ln\left(a\right)+b\ln\left(t\right)-ct. \end{array}$$

The milk yield up to week “*t*” is given by *Y*_*t*_ = *a*∫_0→1_ *t* ^*b*^exp⁡(−*ct*)*dt*.

## 3. Results

**Figure 1:**
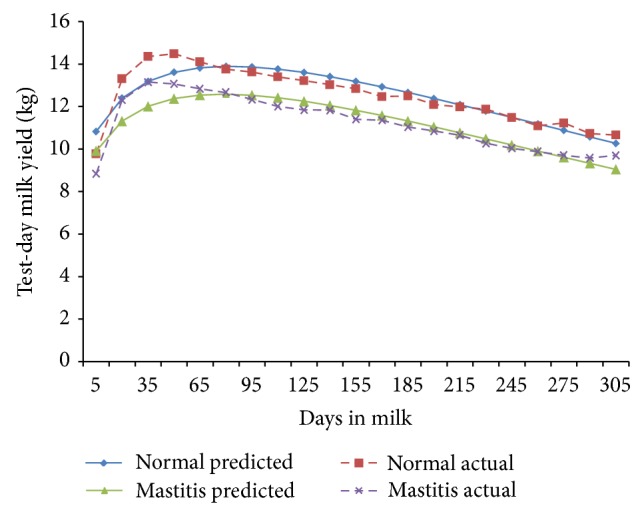
Comparison of the actual and predicted FTDMY of normal and mastitic Karan Fries cows in the first lactation.

**Figure 2:**
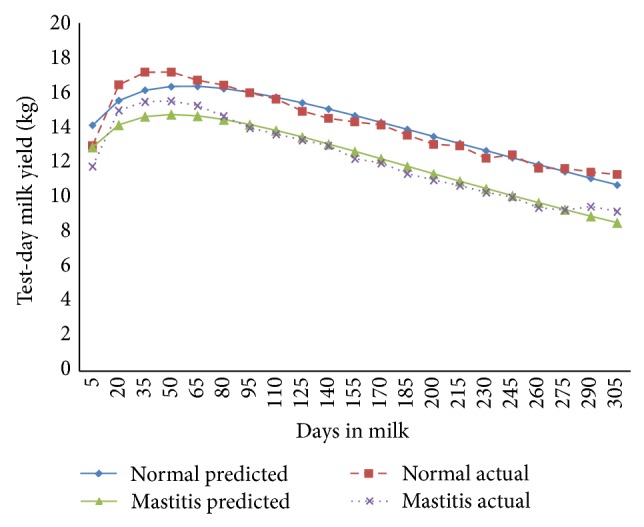
Comparison of the actual and predicted FTDMY of normal and mastitic Karan Fries cows in the second lactation.

**Figure 3:**
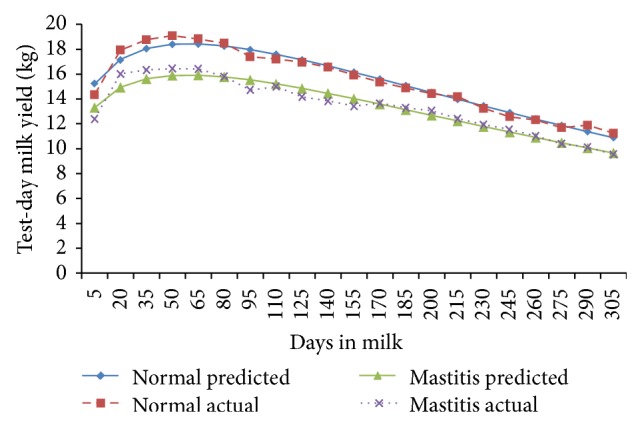
Comparison of the actual and predicted FTDMY of normal and mastitic Karan Fries cows in the third lactation.

**Figure 4:**
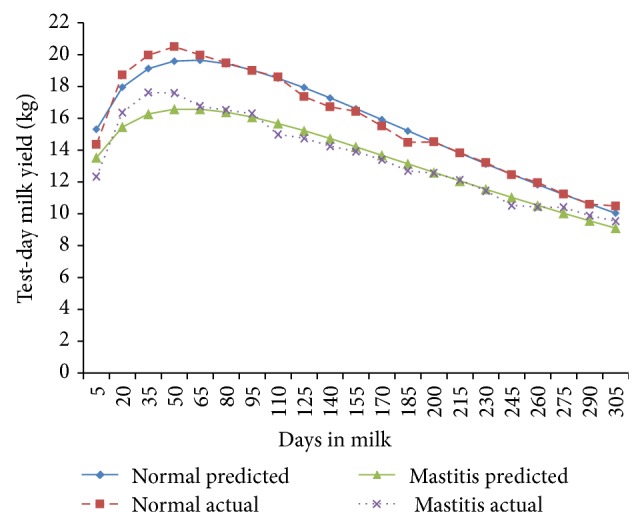
Comparison of the actual and predicted FTDMY of normal and mastitic Karan Fries cows in the fourth lactation.

**Figure 5:**
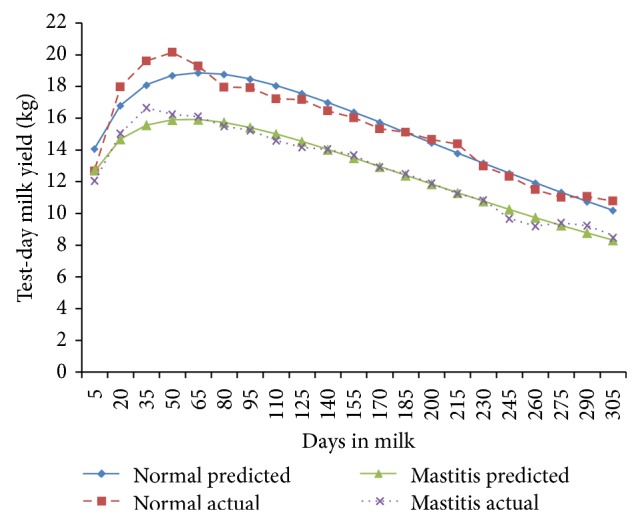
Comparison of the actual and predicted FTDMY of normal and mastitic Karan Fries cows in the fifth and above lactation.

The fortnightly test-day milk yield was less in TD-1 (9.78 ± 0.29 kg/d) and increased (*P* < 0.01) gradually to attain peak in TD-3 and TD-4 (14.34 ± 0.38 and 14.48 ± 0.37 kg/d) in normal cows; however, in mastitis cows, the peak milk yield was attained in TD-3 (13.15 ± 0.37 Kg/d) and was maintained only for this period (Figure 1). The cows of both of the groups exhibited a steady decline (*P* < 0.01) in milk yield with increase in fortnights of lactation. In general the fortnightly test-day milk yield increased till TD-3 and declined steadily with advancement of lactation. In the second parity the FTDMY showed an increasing trend from TD-1 to TD-4 and decreased thereafter gradually in both of the groups. A minimum FTDMY of 11.31 ± 0.39 kg/d and 9.18 ± 0.44 kg/d and a maximum FTDMY of 17.19 ± 0.44 kg/d and 15.53 ± 0.46 kg/d were observed on TD-4 in both of the groups (Figure 2). The minimum FTDMY ranged from 11.25 ± 0.46 kg/d to 9.55 ± 0.47 kg/d in TD-21 and the maximum FTDMY of 19.08 ± 0.53 kg/d and 16.44 ± 0.47 kg/d was observed in TD-4 in normal and mastitis KF cows. However an increase in FTDMY was observed from 14.36 kg and 12.34 kg in TD-1 to a peak yield of 20.50 ± 0.59 kg/d in TD-4 and 17.63 kg/d in TD-3 which subsequently declined (*P* < 0.05) to 10.50 ± 0.54 kg/d and 9.54 ± 0.54 kg/d in TD-21 in normal and mastitis KF cows (Figure 3). The FTDMY milk yield in the 4th parity was maximum in comparison to the first, second, and third parity in both normal and mastitis cows (Figure 4, Table 2). An increase in milk production was observed up to the 4th fortnight and milk productions decreased (*P* < 0.01) during the 5th parity in normal cows. In mastitis cows increase in milk yield was noticed during the first three fortnights and milk production decreased subsequently (*P* < 0.05) until the end of lactation (Figure 5).

Estimation of lactation curve parameters for prediction of fortnightly test-day milk yield.

The parity-wise estimated lactation curve parameters for FTDMY indicated higher average initial milk yield (*a*) in normal cows as compared to mastitis cows in different parities (Table 1). The peak milk yield was observed in the 4th parity (16.45 kg and 14.42 kg) while minimum yield was observed in the 1st parity in both normal and mastitis cows (11.28 versus 10.35 kg/d). Lactation curve parameter of ascending slope parameter up to peak yield (*b*) was similar in both the groups in the 1st parity, declined in the 2nd parity, and increased in the subsequent parities. The descending slope parameter (*c*) was lower in the 1st parity (0.041) and higher in the 4th parity (0.072) in normal cows, but, in mastitis cows, it was minimum in the 1st parity (0.043) and maximum in the 5th and above parity (0.070). Further higher persistency of lactation was observed in the 1st parity (54.34 and 51.40) which declined till the 5th and above parity in all of the cows. The fortnights of peak milk yield ranged from averagely 4.53 to 6.15 fortnights in normal cows and from 4.49 to 5.88 fortnights in mastitis cows. The cows in the first parity took more time (fortnight) to attain peak yield in comparison to subsequent parity; however, peak milk yield increased (*P* < 0.05) with increase in number of parity in both of the groups. The peak yield was minimum in the 1st parity (13.89 kg/d and 12.58 kg/d) and maximum in the 4th parity (19.67 kg/d and 16.61 kg/d) in normal and mastitis cows. The coefficient of determination of variation (*R* ^2^) ranged from 84.88% to 98.18% in normal cows and from 83.93% to 97.68% in mastitis cows during different parities. The RMSE value was minimum in the 3rd parity (0.09 kg) and maximum in the 5th and above parity (0.16 kg) in normal cows; however, in mastitis cows RMSE was minimum in the 5th and above parity (0.09) and maximum in the 4th parity (0.13 kg).

## 4. Discussion

The pattern of change in the FTDMY observed in this study was in agreement with the earlier reports in normal cattle and buffaloes [12–17]. However comparable literature on fortnightly test-day milk yield (FTDMY) and fortnightly average daily milk yield (FADMY) in mastitis crossbred KF cows is scanty. The peak milk yield attained during the 3rd and 4th fortnights in both of the groups corroborates with the values reported earlier in cows [10, 18, 19]. The trend in FTDMY in both of the groups suggested that peak yield duration is not affected by incidence of mastitis in spite of significant decline in milk yield (*P* < 0.01). The lower initial milk yield (*a*) in the first parity and an increasing trend in subsequent parity have also been reported in normal KF cows [10] and Butana dairy cows [20]. Many research findings indicated an increase in initial milk yield value with increase of parity order and the highest initial milk yield reaches the 5th lactation in Friesian and Ayrshire crossbred and KF cows [21–25]; however, such trend was evident up to the 4th parity in this study. The lower initial yield, peak milk yield, and greater persistency in the first parity than the subsequent parity in KF cows as observed in this study corroborate earlier reports [6, 26–31]. This is expected because lactation curves of the first parity are characterized by a less peak milk production and a greater persistency in ruminants [11, 20, 24]. The decline in persistency of lactation with increase in parity order is attributed to age factor as older animals start their lactation at a higher level and had a rapid rate of decline in milk production due to regression of alveolar cells with advancement in age. Further, first-calving animals have less body weight and maturation of mammary tissue is still active, which counterbalances decline in milk production [32]. During the first lactation, animal encounters unfamiliar situations, including the atmosphere of the milking parlor, presence of dairy farmer, and the milking procedures [24]. Thus variation in the initial milk yield in different parities could be due to addition of more number of alveolar cells (secretory cells) at each successive pregnancy which reach their maximum numbers at about the 5th calving and diminish gradually thereafter [33].

The value of ascending slope parameter up to peak yield (*b*) in the present study was lower than reported earlier in different parity of Friesian and Ayrshire crossbred cows due to difference in milk yield of cows, management practices followed in a farm, and breed difference [33]. The similar pattern of increase in constant “*b*”with advancement of parity in both of the groups was also reported in Butana dairy cows [23], HF cows [34], and crossbred cows [35]. However, value of parameter “*b*” was lower than the reported values in HF, KF, and Sahiwal cows [10, 21, 36] than the value of parameter “*b*” observed in our study. The higher descending slope parameter (*c*) observed in this study was in agreement with the findings of previous reports [33, 35] in crossbred cows and was lower than reported values in Sahiwal cows [36] and Holstein cows [21]. The more flat shape of parameter “*c*” in the first parity than the rest of the parities indicated better utilization of feeds and less susceptibility of cows to metabolic and reproductive disorders [37]. The marginal differences in parameters “*a*,” “*b*,” and “*c*” in mastitis cows rather than the normal ones were due to disturbed milk secretion and apoptosis of mammary secretory cells that is solely responsible for the decline in milk yield after peak lactation [38, 39]. It has been found that turn of lactation curve was unimportant on the level of persistency of lactation in Brown Swiss cows [40]. Further occurrence of atypical shapes of lactation curve characterized by the absence of the lactation peak varied from 25 to 42% [41]. Previous report also supports the fact that peak yield is lower in the first parity and increases in subsequent parity [33]. The lower initial and peak milk yield in the first parity and more time to attain peak yield in the first parity than the remaining parities were in agreement with previous reports in Norwegian mastitis cows [25, 33]. It has been found that Holstein x Zebu cows require 71 days to reach peak yield, which was less than the predicted 6.15 fortnights observed in crossbred cows [42]. However, predicted fortnightly peak milk yield was nonsignificantly different in both mastitis and normal cows (5.88 versus 6.15 kg/d). Further moderate to high heritability estimates for different lactation curve shape parameters suggest that these traits can be included in selection schemes [43]. It has been reported that the milk secretory tissue requires more time for peak activity in primiparous cows than multiparous cows due to reduced udder size, less digestive capacity, and directs partitioning of nutrients [24, 41]. The high persistency is associated with a slow rate of decline in milk yield after peak production, while low persistency is associated with a rapid rate of decline in milk yield due to less feed intake [44]. Further persistent lactations are characterized by lower peak yield [37] and reduced metabolic stress in early lactation [45]. The high score of goodness of fit (*R* ^2^ value) of gamma type function corroborates the earlier reports in cows [10, 19, 21, 22, 36]. The gamma function has been reported as the best model because of the small error variance and high determination coefficient [4]. From gamma function, approximately one third (31.28% percent) of lactation curves were named atypical. Atypical curve percentages were found as follows: 44.19% downhill, 45.08% concave, 7.32% LnA negative, and 3.39% C negative. Further first lactation goodness of fit in normal and mastitis cows indicated more fitness of the model in KF crossbred cows as evident from low RMSE value. The latter confirms accuracy of gamma function model to predict milk yield of mastitis cows also [11, 46]. The similar value of *R* ^2^ in normal and mastitis cows further indicated accuracy of model level [31]. The similar RMSE values in mastitis and normal KF cows during different parities also support this fact.

## 5. Conclusion

The lactation curve parameters for fortnightly test-day milk yield exhibited similar trend in normal and mastitis infected cows by attaining peak milk yield in the 3rd and 4th TD followed by a gradual decline in milk yield till the end of lactation. The steeper decline in descending slope (*c*) and increase in initial FTDMY due to steeper rise in ascending slope from the 1st parity onwards could be used as a marker of persistency during different parities in cows. Further, higher *R* ^2^ and lower RMSE confirm the validity of gamma type function in predicting the milk yields in cows suffering from mastitis.

## Figures and Tables

**Table 1:** Parity-wise lactation curve parameters (gamma type function) for prediction of fortnightly test-day milk yield (kg) in Karan Fries crossbred cows.

Karan Fries cows										
Parameters	1st parity		2nd parity		3rd parity		4th parity		5th and above parity	
	Normal cows	Mastitis cows	Normal cows	Mastitis cows	Normal cows	Mastitis cows	Normal cows	Mastitis cows	Normal cows	Mastitis cows
*a*	11.28	10.35	14.78	13.57	16.08	14.03	16.45	14.42	15.09	13.57
*b*	0.254	0.255	0.202	0.213	0.259	0.243	0.333	0.281	0.358	0.310
*c*	0.041	0.043	0.045	0.053	0.056	0.053	0.072	0.063	0.070	0.070
Persistency	54.34	51.41	41.96	35.30	37.59	38.50	33.51	34.82	36.56	33.67
Fortnight of PMY	6.15	5.88	4.53	4.02	4.61	4.58	4.64	4.49	5.07	4.55
Fortnightly PMY (kg)	13.89	12.58	16.39	18.04	18.44	15.93	19.67	16.61	18.85	15.94
*R* ^2^ value (%)	84.88	83.93	93.03	94.87	97.43	95.42	98.18	95.52	94.84	97.68
RMSE (kg)	0.08	0.11	0.12	0.11	0.09	0.11	0.10	0.13	0.16	0.09

(*a* is the initial milk yield after calving; *b* is the ascending slope parameter up to peak yield; *c* is the descending slope parameter; RMSE is the root mean square error; *R* ^2^ is the coefficient of determination of variation; and PMY is the peak milk yield).

**Table 2:** Overall actual and predicted milk yield showing accuracy of the model in crossbred cows.

Parity	Normal cows			Mastitis cows		
	Act.	Pred.	Accuracy %	Act.	Pred.	Accuracy %
1	12.47^a^	12.46^a^	99.02	11.20^a^	11.19^a^	99.90
2	14.13^b^	14.12^b^	99.04	12.20^b^	12.18^b^	99.02
3	15.39^c^	15.39^c^	99.09	13.40^c^	13.39^c^	99.04
4	15.69^c^	15.68^c^	99.06	13.55^c^	13.55^c^	100.00
5 and above	15.32^c^	15.30^c^	99.90	12.79^d,b^	12.79^d,b^	100.00

Superscripts a, b, and c differ significantly (*P* < 0.05) in row and column.
